# *Cacopsyllapruni* (Hemiptera, Psyllidae) in an apricot orchard is more attracted to white sticky traps dependent on host phenology

**DOI:** 10.3897/BDJ.10.e93612

**Published:** 2022-11-16

**Authors:** Dominika Bodnár, Sándor Koczor, Gábor Tarcali, Miklós Tóth, Péter G. Ott, Gergely Tholt

**Affiliations:** 1 Centre of Agricultural Research, Department of Pathophysiology, Budapest, Hungary Centre of Agricultural Research, Department of Pathophysiology Budapest Hungary; 2 Centre of Agricultural Research, Department of Applied Chemical Ecology, Budapest, Hungary Centre of Agricultural Research, Department of Applied Chemical Ecology Budapest Hungary; 3 University of Debrecen, Faculty of Agricultural and Food Sciences and Environmental Management, Institute of Plant Protection, Debrecen, Hungary University of Debrecen, Faculty of Agricultural and Food Sciences and Environmental Management, Institute of Plant Protection Debrecen Hungary; 4 Centre of Agricultural Research, Department of Zoology, Budapest, Hungary Centre of Agricultural Research, Department of Zoology Budapest Hungary

**Keywords:** vector monitoring, plant alternation, host selection, migration, early warning

## Abstract

The colour preference of the plum psyllid, *Cacopsyllapruni* (Hemiptera, Psyllidae), is yet poorly studied. This species is the only known vector of the ‘*Candidatus* Phytoplasma prunorum’, the agent of European stone fruit yellows (ESFY), a devastating disease of several cultivated *Prunus* species in Europe. As ESFY is still uncurable, vector control, thus vector monitoring, is pivotal to protect these trees. *Cacopsyllapruni* is a univoltine, host-shelter-alternating species; overwintered adults migrate from conifer to wild or cultivated *Prunus* species (family Rosaceae) in late winter-early spring. To select the most effective colour indicating the arrivals of the immigrants, yellow, fluorescent yellow, white, red and transparent sticky traps were deployed in an apricot orchard in Hungary. The two most abundant species in sticky traps were *C.pruni* and *C.melanoneura*. Catches of white traps were significantly biased towards *C.pruni* as compared to *C.melanoneura* specimens. Moreover, white sticky traps were better at catching plum psyllids than the other colours. Attraction to white was strongest when immigrants from shelter plants kept arriving in the orchard, coinciding with the blooming principal phenophase of apricot trees. When the host flowering growth stage was over, catches of *C.pruni* in white traps declined sharply to the level of yellow traps that was highest during this post-blooming period. We recommended white sticky traps for promptly monitoring *C.pruni* in apricot orchards because it is more potent and more selective than yellow ones during the critically important early flowering interval.

## Introduction

Jumping plant lice or psyllids (Hemiptera, Psylloidea) are small, phytophagous insects with an elongated body, short antennae and piercing-sucking mouthparts enabling them to feed from phloem sieve elements – and inadvertently inoculation/transmission phloem-dwelling bacterial pathogens from/to this hidden plant compartment. Phloem-dwelling pathogens, such as phytoplasmas and ‘*Candidatus* Liberibacter‘, spread via hemipteran insect vectors in nature (Moreno et al. 2021). With nine species involved, the *Cacopsylla* genus (Psyllidae family) is unique amongst psyllids due to the capability to vector phytoplasmas of the group 16SrX. All nine breed on fruit tree hosts of the family Rosaceae (Jaraush et al. 2019).

The immense economical impact of phytoplasma diseases of these stone fruit, apple and pear trees underlines the importance of detailed knowledge about the life cycle of *Cacopsylla* vector species. One key aspect of their life cycle is whether the psyllids require a shelter plant in winter or overwinter in egg or immature stage (like *Cacopsyllabidens* Šulc, 1907). While the multivoltine *Cacopsylla* species stay on or near their host plant in winter, the univoltine, phytoplasma-vectoring species (*Cacopsyllamelanoneura* (Förster, 1848), C. (Thamnopsylla) pruni (Scopoli, 1763), *C.picta* (Foerster, 1848) and *C.pyrisuga* (Förster, 1848)) are migratory (Hodkinson 2009): the immatures develop from freshly-laid eggs on *Prunus* (Rosaceae) host plants; then, the adults of the new generation migrate to coniferous species in summer to spend the winter; next spring, the overwintered adults leave their shelter and migrate to host plants to breed on them.

Dispersal to a very different habitat (overwintering vs. host plants) is associated with changes in psyllid physiology and ecology, a rare and poorly documented phenomenon amongst psyllids. The reasons for it are not clear, but the observation that the full development of *C.pruni* immatures is not supported by the conifers’ phloem sap (Gallinger and Gross 2020) promotes our understanding of why migration to host plants is obligate.

The psyllids’ flight orientation most probably depends on both olfactory and visual cue ranges during migration, as in other hemipterans (Döring 2014, Bian et al. 2020). Visual stimuli could act as an indicator of host plant patches at long or mid-ranges, whereas selecting an individual host plant or a feeding site is based on chemical stimuli (Bukovinszky et al. 2005, Farnier et al. 2015). Plant volatiles were shown to direct the migratory behaviour of *C.picta* (Mayer and Gross 2007, Mayer et al. 2011, Gross 2016). Phytoplasma-infected host plants were even more attractive than healthy ones (Mayer et al. 2008a, Mayer et al. 2008b).

*Cacopsyllapruni*, the main focus of the present study, is a complex consisting of A and B cryptic species (Sauvion et al. 2007, Peccoud et al. 2013) and is the only known vector of ‘*Candidatus* Phytoplasma prunorum’ (Carraro et al. 1998), a bacterium causing European stone fruit yellows (ESFY), a devastating disease of apricot, Japanese plum, Japanese cherry, peach and almond (Kison and Seemuller 2001). Both A and B can transmit the pathogen (Marie-Jeanne et al. 2020). In Hungary, only B was detected (Mergenthaler et al. 2017, Lepres et al. 2018, Sauvion et al. 2021). Its host plants are only *Prunus* species. *Cacopsyllamelanoneura* transmits ‘*Ca.* Phytoplasma mali’, the agent of apple proliferation in some, but not all, geographical areas (Tedeschi et al. 2002).

*Cacopsyllapruni* and *C.melanoneura* are very close in terms of their life cycle and vectoring capabilities, such as long effective latency (the period needed from the acquisition of the pathogen to become infective) (Thébaud et al. 2009, Candian et al. 2020). The long latency has a consequence that most phytoplasma transmission is performed by overwintered adults. Further details of disease spread also underline the importance of wild hosts, infected nursery material and that spread of a particular pathogen genotype occurs within 50 km (by *C.pruni*, Marie-Jeanne et al. 2020).

Phytoplasmas are obligate parasitic bacteria causing severe diseases and yield losses in various plant cultivars. They are phloem-restricted pathogens transmitted by hemipteran insects (Moreno et al. 2021). Phytoplasma infections are not curable yet; consequently, prevention against them is based on either resistance breeding or vector suppression by physical barriers or chemical agents against its vector (Paleskić et al. 2017, Childers et al. 2022). The efficiency and success of chemical protection against the vectors highly depend on the timing, for which vector monitoring and early warning are necessary (Perring et al. 1999). Based on detailed spatial distribution data, Marie-Jeanne et al. (2020) suggest that psyllid vectors should be controlled during the key period of their return migration after overwintering. Monitoring the arrival of these insects allows growers to implement prophylactic measures against the vectored disease.

There are several methods to monitor the population dynamics of psyllids: entomological net (Drohojowska et al. 2013), branch beating (Tedeschi et al. 2002, Weintraub and Gross 2013), sentinel plants, suction traps, Malaise traps, yellow water trays (Hodkinson 1989), sticky traps (Tedeschi et al. 2002) and clear sticky traps (Weintraub and Gross 2013). To be practical, the standpoint of growers should be considered. The classical branch beating, although effective and non-destructive, is not a good option because it is laborious and sensitive to current weather conditions, such as wind and rain. Sentinel plants are subject to damage and difficult to time. The more sophisticated Malaise and suction traps are expensive and prone to dysfunction, theft and vandalism (Uhler et al. 2021). Sticky traps without a volatile attractant are not very effective, but easy to set up and standardise; effects of weather extremes are average over time and the traps are cheap and passive. Sticky trapping can be used to collect specimens of both sexes and is suitable not only for detecting the presence of an insect in a particular area, but also for tracking its variation in density and migration dynamics (Krüger and Fiore 2019).

To estimate the insect migration dynamics for plant protection, identifying the most attractive colour of sticky traps (without attractant) for the pest is pivotal for adequate and efficient protection measures, especially in the case of vectors of plant pathogens (Hall et al. 2010). As in the case of nearly all hemipterans, the psyllids’ presence is often monitored by yellow sticky traps (Hall et al. 2010).

These insects usually feed on green plant organs, as they suck plant sap from the transport tissues of leaves and shoots (Moreno et al. 2021). Yellow colour is a well-known “supernormal stimulus” of plant parts (Krysan and Horton 1991, Döring and Chittka 2007). Yellow sticky traps are also widely, but not exclusively, used to capture *Cacopsylla* species, including *C.pruni* (Sabaté et al. 2007) and *C.melanoneura* (Tedeschi et al. 2002) or other psyllid species (e.g. transparent and yellow - Krysan and Horton 1991, Brown et al. 2009, Weintraub and Gross 2013, Sabaté et al. 2016).

In this study, we evaluated the attractiveness of five different colours (yellow, white, red, fluorescent yellow and colourless/transparent) to these pests, focusing on the overwintered individuals at the beginning of the immigration period into the orchards to support the timing of plant protection in apricot orchards.

## Materials and Methods

### Experimental design

Our study was conducted in an apricot orchard (*Prunusarmeniaca* L., a mix of several cultivars of different ages) near Boldogkőváralja, Hungary (48°21′00.8″N 21°13′44.3″E). The orchard is mostly surrounded by agricultural fields and natural hedges, where wild blackthorn (*Prunusspinosa* L., host plant of *C.pruni*) and hawthorn (*Crataegusmonogyna* Jacq., host plant of *C.melanoneura*) are common and dominant woody species. A small portion of the orchard’s margin is a mixed forest patch composed of several deciduous and coniferous species. Although the orchard is a plantation where all the trees have reached the age of yield, it was not treated with any pesticides in the previous three months and during the study in 2020.

Commercially available sticky traps (“SZ” series, 10 × 16 cm, produced by CSALOMON®, Plant Protection Institute, CAR, Budapest, Hungary) were deployed for the survey. We used five colours, yellow, fluorescent yellow, red, white and transparent (unpainted control), to test the sticky traps' efficiency in catching psyllids and their potential to promptly catch the first immigrants and monitor the timing of psyllid arrivals; reflectance spectra of traps was previously described (Suppl. material 1; Rőth et al. 2016).

For each trap, two coloured cards (of the same colour) were fastened together back-to-back by metal wires, with sticky surfaces outwards. Each trap was fixed on the branches of apricot trees at 1.5 m above the ground. The minimum distance between traps was 10-15 m in every direction across the orchard and the minimum distance of any trap to field margins was 15 m. In the survey setup, five differently-coloured (yellow, white, red, fluorescent yellow and colourless/transparent) traps were placed in random order in a row, parallel to orchard tree rows, in ten repetitions, the total number of traps being 50 (ten traps of each colour). All traps were checked regularly at 2-3 day intervals, while the actual phenophases of apricot trees were also recorded. When the trap conditions made it necessary (reduced adhesion capacity due to, for example, dust or leaves), all traps were replaced simultaneously within one day, each with the same colour. Based on previous experience, from mid-March, the presence of *C.pruni* individuals in the orchard and the surrounding hedges was checked daily by visual observation of the branches and indicator sticky traps (one trap of each of the five colours). The trapping period started on the day of the appearance of the first *C.pruni* individual, i.e. on 25.03.2020 and lasted 11 weeks (the end of immigration). The traps were replaced six times during the survey on the following dates: 01.04.2020, 07.04.2020, 15.04.2020, 29.04.2020, 08.05.2020 and the traps were collected at the end of day 06.06.2020.

Psyllid specimens were counted and identified according to the keys of Ossiannilsson (1992), whenever the traps were replaced. The traps collected *C.pruni* (both new generation and overwintered individuals), *C.melanoneura* and other psyllid species as well.

Although females of *C.melanoneura* and *C.affinis* (Löw, 1880) psyllid species cannot be identified, based on their morphology, we have not found male individuals of *C.affinis* in our traps. *Cacopsyllamelanoneura* has been recorded (Kontschán et al. 2022) much more frequently than *C.affinis* (Horváth 1885). Additionally, *C.melanoneura* occurs on apricot as well (Jarausch et al. 2009, Lethmayer et al. 2011); thus, we are convinced that we identified female individuals of *C.melanoneura* during our study.

Based on the abundance of each species, we identified adults of the two most frequent species, namely *C.pruni* and *C.melanoneura*. Any other members of the *Cacopsylla* genus, thus, other species and specimens that were not identifiable due to affected condition by glue damage or not yet completed body pigmentation, were regarded as “other *Cacopsylla* spp.”.

### Statistical methods

To test the effect of colours on psyllid catches, we summed up the number of caught specimens by colours by repetitions during the whole observation period. The distributions of the response variables and their residuals were identified by QQ plots, data being transformed when the distributions of response variables were different from the normal distribution. Best-fitting statistical models were selected, based on AIC values and/or by ANOVA. The total number of caught *C.pruni* was logarithmically transformed, then we fitted generalised least squares (GLS) models (R package “nlme”) (Pinheiro et al. 2022). We fitted the GLS model to the total number of caught *C.melanoneura* without data transformation. We performed pairwise tests using Tukey-adjusted P values in the R package “EMmeans” when we compared the total number of catches on every colour within both species (Searle et al. 2012). We compared the aggregate number of *C.pruni* and *C.melanoneura* individuals in yellow traps after square root transformation and in white traps after logarithmic transformation, by fitting GLS models.

Based on the results, we distinguished a sub-period during the survey, called the main immigration period (IM), when the newly-arrived adults were in the highest number. We think that, after this period, most overwintered adults come from the near bushes and not from the conifers. IM lasted from 25.03.2020 (day 0 of the whole observation period) to 15.04.2020 (day 20). Just as for the whole period, we summarised the number of caught specimens on each colour by rows/replications for IM. We fitted GLS models after square root transformation on the number of *C.pruni* individuals in white and yellow traps and on the number of *C.pruni* and *C.melanoneura* individuals caught by white traps, during IM. The catches of *C.melanoneura* in white and yellow traps during IM were compared by the GLS model after logarithmic transformation. All statistical procedures were done with R (#R Studio 1.4, R Core Team 2016, R) and for data visualisation, we used R and JMP (16.1.0, SAS Inc.).

## Results

In 2020, psyllids were captured for 11 weeks from March to June on apricot trees by sticky traps. We identified 1517 psyllids in the Psyllidae family (Suppl. material 2). The majority of the specimens were *Cacopsylla* species, while the second most numerous group was Triozidae with only 33 caught individuals. There were no side-catches of other insect families or species in notable numbers.

**Figure 1.**
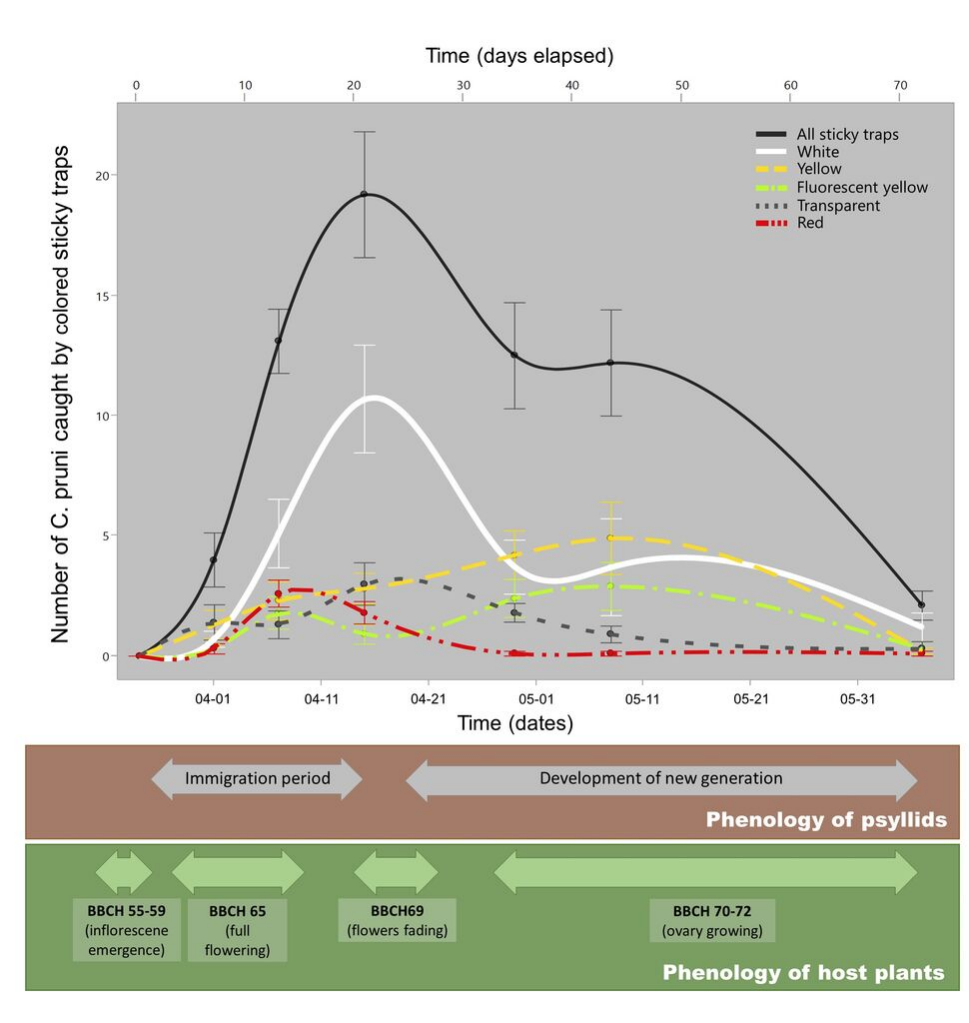
The mean number of *C.pruni* adults caught by sticky traps of different colours during the complete observation period. The catches were summarised across all colours (black line) or within colours (coloured lines) for trap replacement periods and the means were calculated from 10 repetitions. Lower boxes indicate phenological stages of *C.pruni* apricot trees, based on field observations during the complete study. Dots represent means with error bars as standard errors. The spline is fitted continuously. The lower X-axis marks dates, the upper one the days passed from the start of the study.

The first overwintered *C.pruni* adults were caught in sticky traps on 29.03.2020 (Fig. 1). The immigration of *C.pruni* peaked during the middle of April (day 22 in Fig. 1). There was no difference between male and female numbers (based on a sample of 300 specimens, approx. 56% of *C.pruni* were females) and we have not observed juvenile specimens in the traps.

The end of the trapping period did not coincide with the emigration of *C.pruni* from the orchard.

Based on direct observation, the first springtime adults with partial or not complete wing pigmentation appeared on 16.05.2020 and, in total, only nine specimens were caught in the traps until the end of the trapping period.

**Figure 2.**
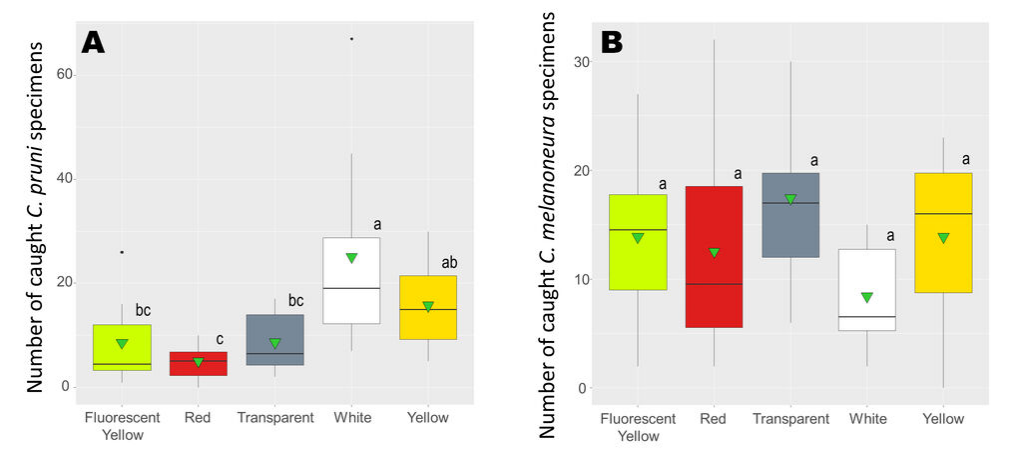
Colour preferences of the two most abundant *Cacopsylla* species in the apricot orchard, *C.pruni* (A) and *C.melanoneura* (B). Graphs show the mean numbers of catches by each coloured sticky trap (Y-axis) during the whole observation period. X-axis lists the trap colours. Horizontal bars represent the medians, vertical bars represent the standard error of means. Statistical means are represented by triangles and interquartile ranges are indicated by boxes and outliers (if present) by black dots. Different letters represent significant differences between colours.

The traps caught a total of 630 overwintered *C.pruni* adults. Aggregate numbers of *C.pruni* adults trapped by distinct colours revealed significant differences between the colours (Fig. 2a). In pairwise comparisons, white traps caught significantly more than red, transparent and fluorescent yellow traps; meanwhile, the catches of yellow traps were significantly higher only against red traps (Suppl. material 3). Although, during the complete observation period, white traps caught the most *C.pruni* individuals, no significant differences were recorded between catches on yellow and white traps. There were no differences in catches between red, transparent and fluorescent yellow traps (Fig. 2a).

**Figure 3.**
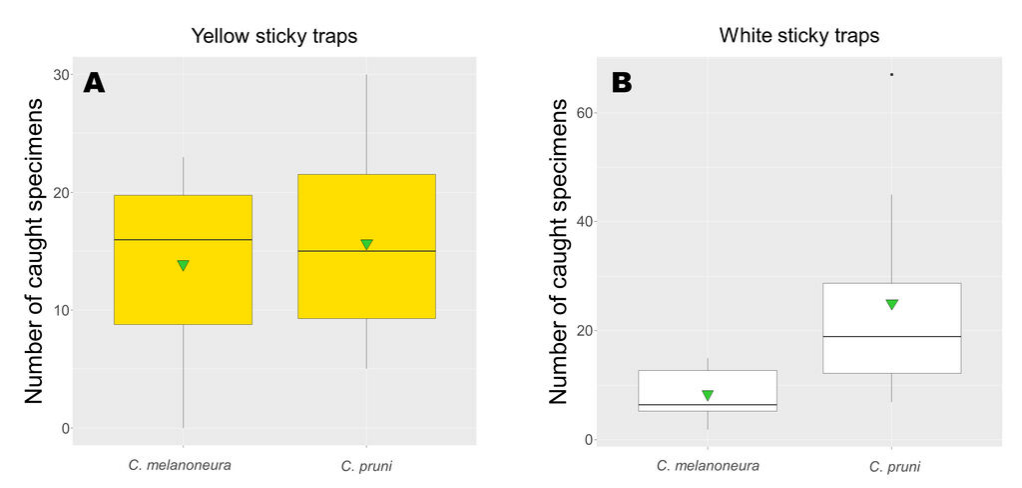
Comparison of the effectiveness of yellow (A) and white (B) sticky traps in catching *C.pruni* and *C.melanoneura* specimens. Graphs show the cumulative catches by each colour during the complete observation period. Horizontal bars represent the medians, vertical bars represent the standard error of means. Statistical means are represented by triangles, interquartile ranges are indicated by boxes and outliers (if present) by black dots.

The most abundant species in the apricot orchard was *C.melanoneura* totalling 661 catches. As for *C.pruni*, we compared the cumulative numbers of *C.melanoneura* adults on the five differently-coloured sticky traps. For this species, the applied trap colours did not influence the catches (Fig. 2b, Suppl. material 3, Suppl. material 4). To detect any difference in colour preference between the two psyllids, we compared the aggregate numbers of *C.pruni* and *C.melanoneura* specimens on white and yellow traps. On the yellow traps, there were no differences between the *C.melanoneura* and *C.pruni* catches, while the white trapped significantly more *C.pruni* individuals than *C.melanoneura* (Fig. 3b) (Fig. 3a, Suppl. material 3, Suppl. material 4).

**Figure 4.**
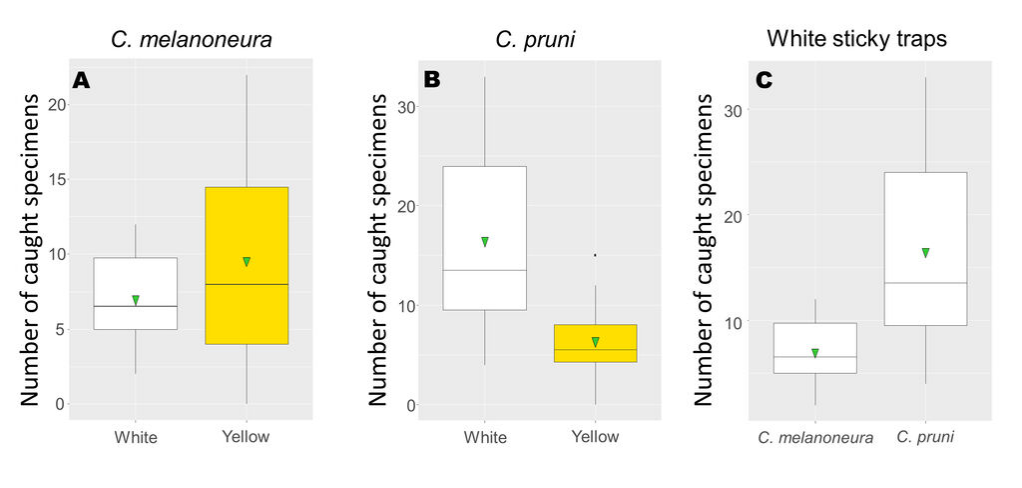
Colour efficiency of sticky traps (means of summed catches) during the immigration period (IM). Comparison of *C.melanoneura* (A) and *C.pruni* (B) numbers on yellow and white traps. Comparison of *C.melanoneura* and *C.pruni* catches on white sticky traps during IM (C). Horizontal bars represent the medians, vertical bars represent the standard error of means. Statistical means are represented by triangles and interquartile ranges are indicated by boxes and outliers (if present) by black dots.

The difference between the catches of *C.pruni* on white and yellow traps was not constant during the whole survey (Fig. 1, Suppl. material 3). White traps caught notably (and significantly) more individuals during the first 3 weeks of observation (immigration period, IM) than yellow ones, while the yellow-coloured traps were more effective after mid-April (Fig. 4).

The period of white catches eminent over those of the other colours corresponded with flowering stages, its increasing part with the mid-stage of full bloom (BBCH 65) of apricot (when flower petals of neighbouring blackthorn became perceptible at BBCH 58) and its decreasing part with the end-stage of flowering (BBCH 69-70) of both plants (Fig. 1). Yellow catches reached peak level - comparable to that of white traps - during foliage expansion, i.e. after petal fall. Thus, to find the best sticky trap colour for timing plant protection treatments, we compared the effect of colours on catches of *C.pruni* and *C.melanoneura* during IM (Fig. 4, Suppl. material 3).

Neither of our coloured sticky traps caught honey bees (*Apismellifera*), although they are responsible for the pollination of the major part of apricot flowers in this orchard and, during the study, a beekeeper operated several colonies in the vicinity of the plantation.

## Discussion

Understanding the behaviour and the life cycle of the *C.pruni* is key to the efficiency and integrated plant protection measures. During the migration of overwintered adults, the visual stimuli could help the univoltine psyllids to find their host plants, although, in the *Cacopsylla* genus, this phenomenon is by far less documented than in the case of Eucalyptus feeding species (Farnier et al. 2015). Additional knowledge of colour preference in *Cacopsylla* species, especially in the case of *C.pruni* and *C.melanoneura*, supports a better understanding of their migration behaviour. Our study provides some additional information to this poorly-documented part of the psyllids’ biology.

White traps attracted *C.pruni* more than yellow or other colours in our test, especially during the immigration period, when overwintered adults appear in orchards. Moreover, the white colour was species-selective, as it caught significantly more *C.pruni* than *C.melanoneura* specimens, even though the latter appeared in higher numbers.

In our survey, *C.pruni* appeared in the highest numbers on white traps when *Prunus* hosts were in the blooming growth stages. At that time, three colours dominate the scenery, that of the host's petals, usually white, the green vegetation on the ground and the dark colours of the bark or ground, which is usually brown(ish) and thought as a colour to be avoided by phytophagous insects (Kostal and Finch 1994). These contrasting colours may favour a scenario where white signals the availability of *Prunus* parts as a food source for *C.pruni*. Psyllids have been known for a long time as part of the aerial plankton (White 1970, Greenslade et al. 2021) and our finding tempts us to assume that colour vision, the preference for white, in particular, helps them find patches of hosts plants in bloom.

Colour preference of overwintered adults may alter after flowering, i.e. when the white colour is depleted. Then, the white colour no longer indicates host plants from any distance. We think that, from that stage, it is more beneficial for psyllids to prefer the freshly occurring green-coloured plant parts instead. This sudden change in phenology-driven plant constitution may have resulted in a sudden change in psyllid behaviour as well (Fig. 1). As insects often prefer and utilise the most abundant food sources (Farnier et al. 2015), the transition from white preference to green one during the emergence of green biomass corresponds with the alternating abundance of white petals and green leaves. Behavioural changes induced by host phenology could serve phenological synchrony between psyllids and their host plants. Thus, phenological synchrony with host plants, especially in the case of host alternation, is key for the long-term fitness of psyllids. Reported examples of such synchrony, manifested through colour preference change of psyllids in parallel with host phenology, are that of *C.pyricola* (Krysan and Horton 1991, Horton and Lewis 1997, Cooper et al. 2012) and three Eucalyptus-feeding psyllids (Farnier and Steinbauer 2016). Our results showed that, after flowering (immigration period), the effectiveness of the white colour decreased to the level of yellow traps. The psyllids involved here presumably had arrived later, no longer directly from conifers, but from neighbouring hedges or they were immigrants having dwelled in the orchard itself for a few weeks. The phenologically synchronised colour preference may also confer a selective advantage to *C.pruni* over competitors, akin to the case of *Eucalyptus*-feeding psyllids (Farnier et al. 2014, Farnier and Steinbauer 2016).

We did not find a specific attractive colour cue for *C.melanoneura*, which might indicate that this species is just drifting amongst apricot trees and probably straying from adjacent hawthorn bushes, as others noted in Austrian orchards (Lethmayer et al. 2011). Moreover, we found here that it was the colourless trap that tended to catch the most from this species, which corresponds with previous results (Mayer and Gross 2007). *Cacopsyllamelanoneura* appeared earlier in the orchard than *C.pruni*. Based on previous results and our findings, we assume that there is a crucial difference in migration behaviour between *C.melanoneura* and *C.pruni*. *Cacopsyllamelanoneura* migrates from a mountainous area to lowland conifers, which means it may search for host plants from closer distances; therefore, it may use mostly chemical cues, rather than visual stimuli, as has been shown previously (Mayer and Gross 2007). Our results support this as we did not find a preferred colour for *C.melanonerua*. On the other hand, *C.pruni* most likely migrates differently (Thébaud et al. 2009, Jarausch and Jarausch 2014): it may fly from winter shelter to summer host directly as aerial plankton (Greenslade et al. 2021). In this case, searching for a landing site with possible host plants could be driven by visual cues. After landing in a habitat, the host plant selection of *C.pruni* is possibly driven by both visual and olfactory cues (Gallinger et al. 2019). This could explain the colour preference and the change of it over time in *C.pruni*.

We found that the two major psyllid pests were *C.melanoneura* and *C.pruni* in a plantation of a key apricot-growing area in Hungary (northern Hungary), in spring 2020. They were reported several times as prominent in apricot orchards in central Europe (Fialová et al. 2004, Jarausch et al. 2009, Lethmayer et al. 2011, Warabieda et al. 2018, Mergenthaler et al. 2017, Riedle-Bauer et al. 2019). With species having so similar phenology, our finding has been predictable. Being quantitative, our results also allowed a comparison of these two species concerning their colour preference during their presence in the orchard.

Considering the above-discussed distinct colour preferences of the two most frequent psyllids in the apricot orchard, we suggest the use of white sticky traps instead of yellow ones in stone fruit plantations to detect the appearance and monitor the migration dynamics of the vector of ESFY phytoplasma, *C.pruni*, during the flowering growth stages. Compared to the popular yellow or other colours, white is more effective, because it will catch earlier and more of the plum psyllid than the other major psyllid species, *C.melanoneura*. This has consequences for plant protection practices, as insecticide sprayings can be better scheduled, improving effectiveness and sparing costs. This intriguing difference between the two genus members suggests previously unknown diversity of jumping plant lice in terms of colour preference. Such species-specific results have not been found in other species within one genus.

## Supplementary Material

7DEFE64E-D0C2-527A-BA8B-1FD204BACEF7 10.3897/BDJ.10.e93612.suppl1 Supplementary material 1 Reflectance spectra of coloured sticky traps in the 275-800 nm wavelength interval. Data type Figure Brief description Measurements made as described in Rőth et al. (2016). File: oo_758191.jpg https://binary.pensoft.net/file/758191 Dominika Bodnár, Sándor Koczor, Gábor Tarcali, Miklós Tóth, Peter G. Ott and Gergely Tholt

8301EEB0-3E02-5B9C-90A1-C68B160BF028 10.3897/BDJ.10.e93612.suppl2 Supplementary material 2 Supplementary Table 1. Summary of *Cacopsylla* individuals caught by coloured sticky traps Data type occurrences Brief description Psyllids were captured during 11 weeks from March to June on apricot trees by different coloured sticky traps. File: oo_725670.docx https://binary.pensoft.net/file/725670 Dominika Bodnár, Sándor Koczor, Gábor Tarcali, Miklós Tóth, Peter G. Ott and Gergely Tholt

140B92C7-4956-5959-8DC8-F2347AB51A90 10.3897/BDJ.10.e93612.suppl3 Supplementary material 3 Supplementary Table 2. Summary of used statistical procedures and their results. Data type Used statistical procedures and results Brief description Used statistical procedures and results. File: oo_725671.docx https://binary.pensoft.net/file/725671 Dominika Bodnár, Sándor Koczor, Gábor Tarcali, Miklós Tóth, Peter G. Ott and Gergely Tholt

B049C7C1-FA68-5B95-80ED-A710E39A2B11 10.3897/BDJ.10.e93612.suppl4 Supplementary material 4 Supplementary Table 3. Summary of statistical results of pairwise comparisons within species by emmeans.
Data type statistical results Brief description Summary of statistical results of pairwise comparisons within species by emmeans. File: oo_725674.docx https://binary.pensoft.net/file/725674 Dominika Bodnár, Sándor Koczor, Gábor Tarcali, Miklós Tóth, Peter G. Ott and Gergely Tholt
