# Supplementary figures and images for: Cacopsyllapruni (Hemiptera, Psyllidae) in an apricot orchard is more attracted to white sticky traps dependent on host phenology
Source: Biodivers Data J. 2022 Nov 16;10:e93612. doi: 10.3897/BDJ.10.e93612 (PMC9836614; doi:10.3897/BDJ.10.e93612)

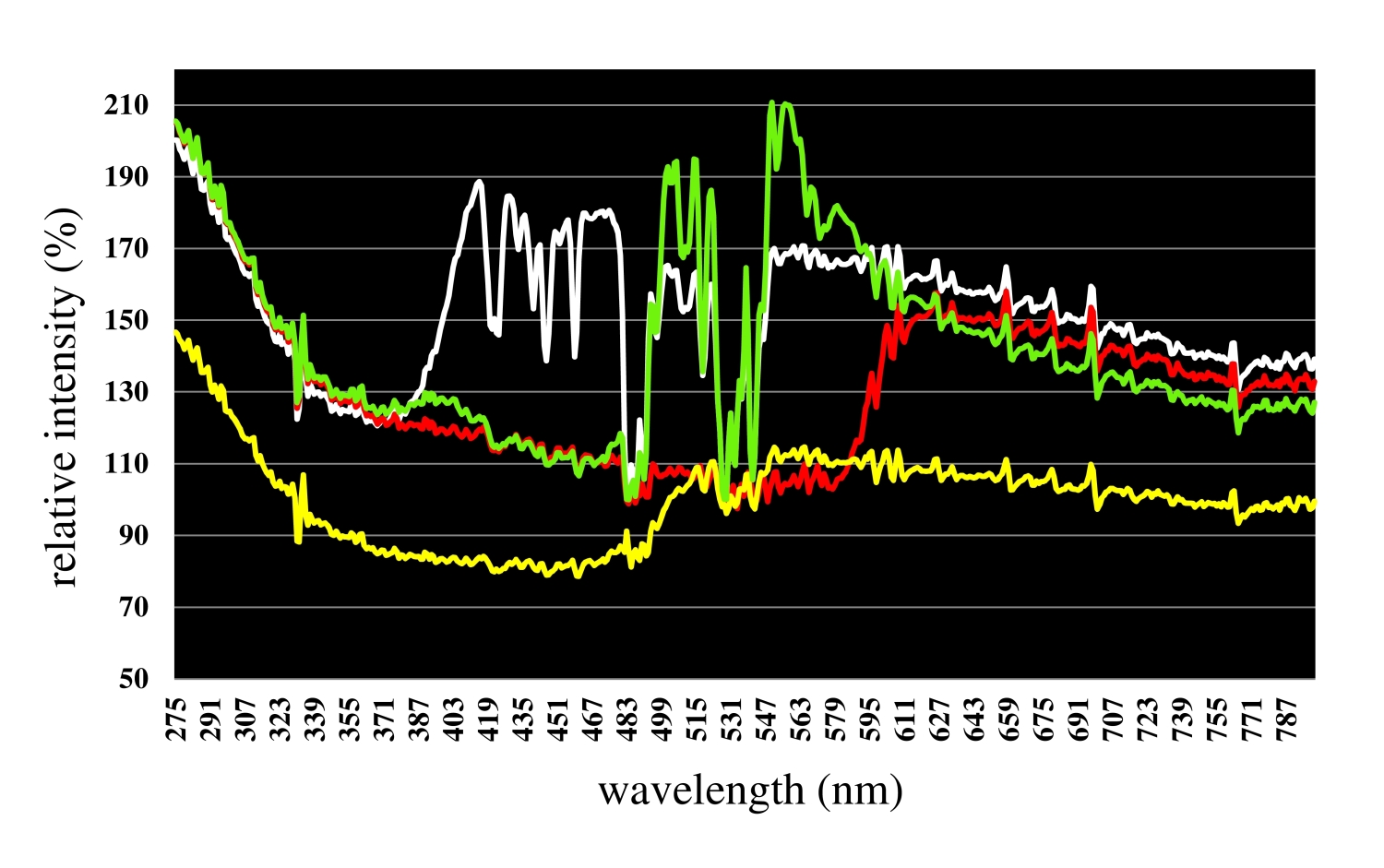

Supplement: Supplementary material 1 — Reflectance spectra of coloured sticky traps in the 275-800 nm wavelength interval. [file bdj-10-e93612-s001.jpg]
