# Supplementary material for: Cacopsyllapruni (Hemiptera, Psyllidae) in an apricot orchard is more attracted to white sticky traps dependent on host phenology
Source: Biodivers Data J. 2022 Nov 16;10:e93612. doi: 10.3897/BDJ.10.e93612 (PMC9836614; doi:10.3897/BDJ.10.e93612)
Supplement: Supplementary material 2 — Supplementary Table 1. Summary of Cacopsylla individuals caught by coloured sticky traps [file bdj-10-e93612-s002.docx]

**Supplementary Table 1. Summary of *Cacopsylla* individuals caught by colored sticky traps**

| Trap colour | Summarized number of *Cacopsylla* spp. individuals | Summarized number of *C. pruni* by colors | Summarized number of *C. melanoneura* by colors | Summarized number of other *Cacopsylla sp*. by colors | Share of *C. pruni* | Share of *C. melanoneura* | Other *Cacopsylla sp.* |
| --- | --- | --- | --- | --- | --- | --- | --- |
| White | 390 | 249 | 84 | 57 | 63.85% | 21.54% | 14.62% |
| Yellow | 367 | 158 | 139 | 70 | 43.05% | 37.87% | 19.07% |
| Fluorescent yellow | 262 | 86 | 139 | 37 | 32.83% | 53.05% | 14.12% |
| Red | 191 | 50 | 125 | 16 | 26.18% | 65.45% | 8.38% |
| Transparent | 307 | 87 | 174 | 46 | 28.34% | 56.68% | 14.98% |
| All | 1517 | 630 | 661 | 226 | 41.52% | 43.57% | 14.89% |
