# Supplementary material for: Cacopsyllapruni (Hemiptera, Psyllidae) in an apricot orchard is more attracted to white sticky traps dependent on host phenology
Source: Biodivers Data J. 2022 Nov 16;10:e93612. doi: 10.3897/BDJ.10.e93612 (PMC9836614; doi:10.3897/BDJ.10.e93612)
Supplement: Supplementary material 3 — Supplementary Table 2. Summary of used statistical procedures and their results. [file bdj-10-e93612-s003.docx]

**Supplementary Table 2. Summary of used statistical procedures and their results.**

| **Multiple comparisons** | | | | | | | | |
| --- | --- | --- | --- | --- | --- | --- | --- | --- |
| Observation period | Subject | Compared variables | | Model | Data transformation | Results of test statistics | d.f. | p - value |
| Full | *C. pruni* | all color | all color | GLS | log | t = 9.23 | 50 | <0.000* |
| Full | *C. melanoneura* | all color | all color | GLS | - | t = 5.68 | 50 | <0.005* |
| **Pairwise comparisons** | | | | | | | | |
| Observation period | Subject | Compared variables | | Model | Data transformation | Results of test statistics | d.f. | p – value |
| Full | White sticky traps | *C. pruni* | *C. melanoneura* | GLS | log | t = 3.49 | 20 | 0.002* |
| Full | Yellow sticky traps | *C. pruni* | *C. melanoneura* | GLS | sqrt | t = 0.68 | 20 | 0.5 |
| Immigration period | *C. pruni* | White | Yellow | GLS | sqrt | t = -3.05 | 20 | 0.006* |
| Immigration period | *C. melanoneura* | White | Yellow | GLS | log | t = 0.15 | 20 | 0.879 |
| Immigration period | White sticky traps | *C. pruni* | *C. melanoneura* | GLS | sqrt | t = 3.04 | 20 | 0.006* |
