# Supplementary material for: Cacopsyllapruni (Hemiptera, Psyllidae) in an apricot orchard is more attracted to white sticky traps dependent on host phenology
Source: Biodivers Data J. 2022 Nov 16;10:e93612. doi: 10.3897/BDJ.10.e93612 (PMC9836614; doi:10.3897/BDJ.10.e93612)
Supplement: Supplementary material 4 — Supplementary Table 3. Summary of statistical results of pairwise comparisons within species by emmeans. [file bdj-10-e93612-s004.docx]

**Supplementary Table 3. Summary of statistical results of pairwise comparisons within species by emmeans.**

| Species | Compared variables (colors) | | t.ratio | d.f. | p - value |
| --- | --- | --- | --- | --- | --- |
| *C. pruni* | White | Fl.Yellow | -3.596 | 45 | 0.0068* |
|  | White | Red | -4.836 | 45 | <0.000* |
|  | White | Transparent | -3.151 | 45 | 0.023* |
|  | White | Yellow | 1.204 | 45 | 0.748 |
|  | Yellow | Fl.Yellow | -2.392 | 45 | 0.136 |
|  | Yellow | Red | -3.632 | 45 | 0.006* |
|  | Yellow | Transparent | -1.947 | 45 | 0.308 |
|  | Fl.Yellow | Transparent | -0.445 | 45 | 0.991 |
|  | Fl.Yellow | Red | 1.240 | 45 | 0.728 |
|  | Red | Transparent | -1.685 | 45 | 0.453 |
|  |  |  |  |  |  |
| *C. melanoneura* | White | Yellow | -1.591 | 45 | 0.51 |
|  | White | Fl. Yellow | 1.591 | 45 | 0.51 |
|  | White | Transparent | 2.603 | 45 | 0.086 |
|  | White | Red | 1.186 | 45 | 0.759 |
|  | Yellow | Fl. Yellow | 0.000 | 45 | 1.00 |
|  | Yellow | Transparent | 1.012 | 45 | 0.848 |
|  | Yellow | Red | -0.405 | 45 | 0.994 |
|  | Fl. Yellow | Transparent | -1.012 | 45 | 0.848 |
|  | Fl. Yellow | Red | 0.405 | 45 | 0.994 |
|  | Transparent | Red | -1.417 | 45 | 0.619 |
